# Development and evaluation of a novel xeno-free culture medium for human-induced pluripotent stem cells

**DOI:** 10.1186/s13287-022-02879-z

**Published:** 2022-06-03

**Authors:** Ying Hua, Kenji Yoshimochi, Junjun Li, Kazuhiro Takekita, Motoshi Shimotsuma, Lingjun Li, Xiang Qu, Jingbo Zhang, Yoshiki Sawa, Li Liu, Shigeru Miyagawa

**Affiliations:** 1 Department of Cardiovascular Surgery, Osaka University Graduate School of Medicine, Osaka, 565-0871 Japan; 2 NACALAI TESQUE, INC. Research and Development Department, Kyoto, 604-0855 Japan; 3 Division of Cardiovascular Surgery, Department of Design for Tissue Regeneration, Graduate School of Medicine, Osaka, 565-0871 Japan; 4 Osaka Police Hospital, Osaka, 543-0035 Japan

**Keywords:** Human-induced pluripotent stem cell, Culture system, Chemically defined medium, Xeno-free, Cost-effective, Self-renewal

## Abstract

**Background:**

Human-induced pluripotent stem cells (hiPSCs) are considered an ideal resource for regenerative medicine because of their ease of access and infinite expansion ability. To satisfy the sizable requirement for clinical applications of hiPSCs, large-scale, expansion-oriented, xeno-free, and cost-effective media are critical. Although several xeno-free media for hiPSCs have been generated over the past decades, few of them are suitable for scalable expansion of cultured hiPSCs because of their modest potential for proliferation and high cost.

**Methods:**

In this study, we developed a xeno-free ON2/AscleStem PSC medium (ON2) and cultured 253G1 hiPSCs on different matrices, including iMatrix-511 and gelatin nanofiber (GNF) in ON2. Over 20 passages, we evaluated cell proliferation by doubling times; pluripotency by flow cytometry, immunofluorescence staining and qRT-PCR; and differentiation ability by three germ layer differentiation in vitro and teratoma formation in severe combined immunodeficiency mice, followed by histological analysis. In addition, we compared the maintenance effect of ON2 on hiPSCs with StemFit® AK02 (AK02N) and Essential 8™ (E8). Besides 253G1 hiPSCs, we cultivated different hiPSC lines, including Ff-l01 hiPSCs, ATCC® ACS-1020™ hiPSCs, and Down’s syndrome patient-specific ATCC® ACS-1003™ hiPSCs in ON2.

**Results:**

We found that 253G1 hiPSCs in ON2 demonstrated normal morphology and karyotype and high self-renewal and differentiation abilities on the tested matrices for over 20 passages. Moreover, 253G1 hiPSCs kept on GNF showed higher growth and stemness, as verified by the shorter doubling time and higher expression levels of pluripotent markers. Compared to AK02N and E8 media, 253G1 hiPSCs grown in ON2 showed higher pluripotency, as demonstrated by the increased expression level of pluripotent factors. In addition, all hiPSC lines cultivated in ON2 were able to grow for at least 10 passages with compact clonal morphology and were positive for all detected pluripotent markers.

**Conclusions:**

Our xeno-free ON2 was compatible with various matrices and ideal for long-term expansion and maintenance of not only healthy-derived hiPSCs but also patient-specific hiPSCs. This highly efficient medium enabled the rapid expansion of hiPSCs in a reliable and cost-effective manner and could act as a promising tool for disease modeling and large-scale production for regenerative medicine in the future.

**Supplementary Information:**

The online version contains supplementary material available at 10.1186/s13287-022-02879-z.

## Background

Human-induced pluripotent stem cells (hiPSCs) were first generated by introducing four reprogramming factors into somatic cells [1, 2]. As a replacement for human embryonic stem cells (hESCs), hiPSCs share the same characterization of unlimited proliferation and efficient differentiation into all three germ layers as hESCs but lack ethical concerns and thus have become a promising source for drug screening, disease modeling, and regenerative medicine [3–9]. The huge demand for high-quality hiPSCs for clinical research has prompted the development of large-scale culture systems. Earlier hiPSC culture systems, combined with mouse embryonic fibroblast (MEF) feeder cells generally supplemented with fetal bovine serum (FBS), could successfully maintain the survival and expansion of hiPSCs [1, 2, 10]. However, the chemically undefined and xenogeneic components contained in culture systems pose pathogenic risks and could lead to immune rejection; the presence of such components also limits our understanding of the underlying molecular mechanisms, which greatly hinders the clinical application of hiPSCs [11–13]. In addition, such culture systems are not cost-effective and lack consistency among batches. To solve these problems, researchers are focusing on optimizing the ingredients of both media and matrix and considerable developments have been made over the past 10 years. The culture systems for hiPSCs have evolved from xenogeneic to homologous and from undefined to defined.

Matrigel, a mixture of extracellular matrix (ECM) derived from mouse Engelbreth-Holm-Swarm tumors, acts as an alternative to MEFs for the maintenance of various cells [14]. Although it works well for maintaining hiPSCs, it is still xenogeneic and undefined and thus not suitable for clinical application [15]. Researchers have started to focus on adhesive proteins contained in Matrigel and synthesized human recombinant ECM proteins. Laminin is a major ECM protein required during embryogenesis, and laminin-based substrates are more efficient than Matrigel or other ECM proteins for the adhesion, survival, and self-renewal of hiPSCs [16, 17]. Various commercialized laminin products, such as E8-fragment of laminin-511 (commercial name iMatrix-511), have been generated and are widely utilized, not only in laboratory studies but also in clinical trials with hiPSCs [18]. Fibronectin (FN) and vitronectin (VN) are also commercialized substrates for hiPSCs; however, the reduced effect of FN and VN has encouraged researchers to focus on more validated matrices such as laminin and other novel substrates [18–20]. Recently, we developed a novel low-attached substrate made up of gelatin nanofiber (GNF), which showed a similar ability to Matrigel for the long-term expansion of hiPSCs [21]. Although GNF has not yet been commercialized, it is a potential candidate for hiPSC cultivation due to its low cost and efficient maintenance [22, 23].

Conventional culture media for hiPSCs are generally made up of basic medium (such as DMEM/F12) supplemented with FBS or animal-derived cytokines [1, 10]. The critical principle for clinical products is to avoid animal-origin or chemically undefined ingredients. The first chemically defined commercial medium for hESCs/hiPSCs is mTeSR^TM^1 medium (mTeSR1, STEMCELL Technologies, USA), which, although being expensive and xenogeneic, has been widely used for routine laboratory studies because of its high proliferative potential [15, 24–26]. Essential 8™ medium (E8, Gibco, USA) is the simplest xeno-free medium for hiPSCs, which was generated by simplifying the excess components in mTeSR1 and retaining only four basic growth factors: insulin, transferrin, basic fibroblast growth factor (bFGF), and transforming growth factor beta (TGFβ) [27]. With its recommended substrate vitronectin-N fragment (rhVTN-N), such a combination is usually applied to the expansion of hESCs/hiPSCs. StemFit® AK02N medium (AK02N, Ajinomoto, Japan) is an albumin-containing xeno-free medium that is made to be used with laminin-511 E8 fragment (LM511-E8) substrate [28]. This AK02N-(LM511-E8) combination is expandable and reproducible and has been widely used for the propagation of clinical-grade hiPSCs [9, 29]. However, the fixed combinations of medium and matrix, such as E8-rhVTN-N and AK02-LN511-E8, are difficult to separate and have limited suitable substrates. Therefore, researchers must use the expensive mTeSR1 when using uncommon substrates. Moreover, none of the media mentioned above are cost-effective enough for large-scale expansion.

Therefore, this study was conducted to develop a xeno-free medium that is compatible with various substrates. We developed a versatile ON2/AscleStem hiPSC medium (shortened to ON2) for hiPSCs, which is xeno-free and compatible with multiple matrices. This medium was effective for the stable maintenance of not only healthy hiPSC lines but also patient-derived hiPSC lines. Coupled with lower cost, the developed xeno-free and chemical ON2 medium holds promise for scaled-up production of hiPSCs for drug screening, disease modeling, and regenerative applications.

## Methods and materials

### Components in ON2

The newly developed xeno-free and chemically defined medium ON2 was manufactured by NACALAI TESQUE INC., JP, and includes a basal medium supplemented with several growth factors. Materials were purchased form Nacalai Tesque Company except as specifically pointed out herein and the catalog number had been provided below. The basal medium is made up of DMEM/F-12 (17155-25) containing a variety of amino acids, vitamins, inorganic salts required for cell survival and provided with l-ascorbic acid (13570-82), selenium (31824-02), insulin (12878-86), and transferrin (12879-34). Basal medium is stored at 4 °C after the osmolarity is adjusted to 340 mosmol and pH to 7.4 with NaHCO_3_ (09655-25). Growth factors such as FGF2 (AF-100-18B, PeproTech), albumin (22000AMX02343, Japan Blood Products Organization), activin A (AF-120-14E, PeproTech), leukemia inhibitory factor (NU0013-1-AF), and chondroitin sulfate (08815-84) are supplemented in ON2 medium and stored below − 20 °C usually and used in combination with basal medium. All ingredients were purified and chemically defined, and no xenogeneic products were included. Media components are described in Additional file 1: Table S1.

### Human pluripotent stem cell lines

Three healthy human iPSC lines, 253G1 (Riken, JP), HLA-homo Ff-l01 (CiRA, JP), and ACS-1020 (American Type Culture Collection (ATCC), Manassas, VA, USA), and a Down Syndrome (DS) patient-specific hiPSC line ACS-1003 (ATCC) were used in this work, and all experiments relative to hiPSCs were performed according to Osaka University guidelines [28, 30]. Prior to the assay, 253G1 hiPSCs generated from normal human dermal fibroblasts were maintained on mitotically arrested MEFs in Primate ESC medium (RCHEMD001, REPROCELL) supplemented with 5 ng/ml human bFGF, according to the manufacturer’s protocol, and subsequently transferred to feeder-free culture systems for prolonged expansion. To confirm the reliability of the developed xeno-free culture system, hiPSC lines ACS-1020, HLA-homo Ff-l01, and DS patient-specific ACS-1003 were used. HLA-homo Ff-l01 hiPSCs were generated from peripheral blood mononuclear cells of healthy Japanese adults with homozygous human leukocyte antigen (HLA) haplotypes, which was regarded as a clinical-use hiPSC line and maintained on iMatrix-511 in E8 medium. The ACS-1020 hiPSC line reprogrammed from healthy adult donor-derived hepatic fibroblasts and DS ACS-1003 hiPSC line generated from foreskin fibroblasts isolated from a newborn Down Syndrome donor were purchased from the ATCC and stored at − 150 °C and directly thawed into iMatrix-511 substrate in ON2 for subsequent expansion under xeno-free conditions.

### Substrate preparation and optimization

253G1 hiPSCs were used for the optimization of culture conditions in ON2. Different concentrations of recombinant human ECM proteins, including truncated recombinant human VN (A14700, Gibco, Thermo Fisher Scientific, Waltham, MA, USA), human plasma FN (063-05591, Fujifilm WAKO, Osaka, Japan), iMatrix-511(892012, Nacalai, Kyoto, Japan), and GNF were evaluated. For FN or VN, a concentration range is provided by the manufacturers. Therefore, we first determined the respective optimal coating concentrations for hiPSC growth. Culture dishes were precoated with FN at 0.5 g/cm^2^, 1 g/cm^2^, or 2 g/cm^2^ or VN at 0.5 g/cm^2^ or 1 g/cm^2^ for 2 h at 37 °C according to the manufacturer’s instructions. Then, 253G1 hiPSCs were cultured for several passages to select the optimal coating condition for both matrices. As iMatrix-511 has a standard optimal coating concentration, we coated plates with iMatrix-511 at 0.5 µg/cm^2^ for 1 h at 37 °C before cell seeding according to the manufacturer’s instructions. Dulbecco’s PBS was used to dilute the ECM proteins mentioned above. For GNF substrate, the gelatin (11 wt%, type B, from porcine skin; Sigma-Aldrich, St. Louis, MA, USA) solution was dissolved in water containing acetic acid and ethyl acetate at a ratio of 3:2 for 16 h, and then GNF was fabricated on glass slides by electrospinning and subsequently cross-linking in ethanol containing 0.2 M *N*-ethyl-*N*′-(dimethylaminopropyl)carbodiimide and 0.2 M *N*-hydroxysuccinimide for 4 h, referring to a previous study [22]. The developed GNFs were rinsed with 99.9% ethanol three times and dried on a bench before use. In this work, GNFs with an electrospinning time of 20 min were used for the prolonged expansion of hiPSCs.

### Long-term expansion and maintenance of hiPSCs

For long-term expansion, hiPSCs were grafted to prepared substrates with ON2 and seeding densities were adjusted to meet the subculture frequency of once a week on iMatrix-511, FN, and VN or twice on GNF. The most used serum-free medium mTeSR1 and xeno-free media E8 and AK02N were used as positive controls in our study. hiPSCs were maintained at 37 °C with 5% CO_2_ and 5% O_2_, and the culture media were changed daily.

Passages were carried out once or twice a week according to matrix conditions, when the cell density reached > 80% of the coating surface. For the subculture, hiPSCs on iMatrix-511, FN, or VN were rinsed once with D-PBS, treated with enzymatic cell dissociation buffer Accumax (17087-54, NACALAI TESQUE, Kyoto, Japan) at 37 °C for 4 min, detached either as clusters for FN and VN, or as single cells for iMatrix-511. In case of cells on GNF, they were treated with enzyme-free PBS-based EDTA cell dissociation buffer (13151014, Gibco, USA) at 37 °C for 4 min after being rinsed once with D-PBS. Collected cells were engrafted on prepared plates with new media. For the first 24 h, 10 µM ROCKi was used to improve the survival of single hiPSCs. This procedure was repeated for at least 10 passages over 2 months before further characterization assessment.

### Cell population doubling time

For cells that allow for single-cell passage, the population doubling time of these cells can be achieved by monitoring the initial number and cell yield over a period of two time points. In this study, hiPSCs cultured on iMatrix-511 and GNF were counted at the end of each passage, and the growth rate of hiPSCs was estimated by calculating the respective doubling time using the online calculator (http://www.doubling-time.com/compute.php?lang=en), as DT is a reflection of proliferation rate.

### Flow cytometry analysis

hiPSCs were dissociated into single cells and washed three times with D-PBS. A total of ~ 2 × 10^5^ cells were stained with the following PE-conjugated antibodies diluted in 500 µL 3% BSA/PBS blocking buffer: anti-SSEA-4 (mouse monoclonal IgG3, 1:250; 330406), anti-SSEA-1 (mouse monoclonal IgG1, 1:250; 323006), anti-TRA-1-60 (mouse monoclonal IgM, 1:500; 330610) or the corresponding isotype controls, all purchased from BioLegend (San Diego, CA, USA). After incubation in the dark at room temperature for 30 min, hiPSCs were washed three times with PBS and assessed by immune-positive cell surface markers using a FACS Canto II flow cytometer (BD Biosciences, Franklin Lakes, NJ, USA). A minimum of 10,000 cell events were acquired during each assay, and the data obtained were analyzed using FlowJo (v10, Tree Star).

### Immunofluorescence staining

Samples were fixed in 4% paraformaldehyde buffer at 20–25 °C for 30 min and permeabilized with 0.5% (v/v) Triton X-100/PBS at 4 °C overnight. The samples were blocked with blocking buffer (5%, v/v, normal donkey serum; 5%, v/v, normal goat serum; 3%, v/v, BSA; and 0.1%, v/v, Tween 20 in PBS) at 20–25 °C for 1 h and incubated with the following anti-human primary antibodies diluted in 0.5% (v/v) Triton X-100/PBS solution at 4 °C overnight: anti-OCT3/4 (mouse monoclonal IgG2b, 200:1, sc-5279, Santa Cruz Biotechnology, Dallas, TX, USA), anti-NANOG (rabbit polyclonal IgG, 400:1, 4903, Cell Signaling Technology, Danvers, MA, USA), anti-β-tubulin III (rabbit polyclonal IgG, 400:1, 5568, Cell Signaling Technology), anti-myosin (mouse monoclonal IgG1, 400:1, M4276, Sigma-Aldrich), and anti-alpha fetoprotein (mouse monoclonal IgG2a, 400:1, A8452, Sigma-Aldrich). Next, the samples were incubated with the following appropriate secondary antibodies diluted in blocking buffer at 20–25 °C for 1 h: Alexa Fluor 488-conjugated goat anti-mouse IgG (1:500, A11029, Life Technologies, Carlsbad, CA, USA) or Alexa Fluor 647-conjugated goat anti-mouse or rabbit IgG (1:500, A21236 and A21245, Life Technologies). Finally, the samples were counterstained with DAPI (40-6-diamidino-2-phenylindole, 1:1000, Wako, Osaka, Japan) at 20–25 °C for 30 min. Samples were washed three times with PBS after each step and protected from light after the addition of secondary antibodies. Visualized images were captured using confocal microscopy (NIKON A1).

### Quantitative reverse transcription-polymerase chain reaction (qRT-PCR)

Total RNA was isolated using TRIzol reagent (Life Technologies) following the manufacturer’s instructions. RNA concentrations were determined using a NanoDrop1000 spectrometer (Thermo Fisher Scientific, Waltham, MA, USA), and complementary DNA (cDNA) was synthesized using a first-strand synthesis kit (TaKaRa, Shiga, Japan) on the SimpliAmp Thermal Cycler (Applied Biosystems, Thermo Fisher Scientific) and then amplified in a 96-well format with SYBR Green PCR Master Mix (Thermo Fisher Scientific) on a StepOnePlus Real-Time PCR system (Applied Biosystems, Thermo Fisher Scientific). The running protocol was as follows: initial denaturation at 95 °C for 10 min, followed by 40 cycles of amplification at 95 °C for 15 s and 60 °C for 70 s. Relative gene expression levels were assessed by normalizing to the reference gene, glyceraldehyde 3-phosphate dehydrogenase (*GAPDH*), using the 2^−ΔΔCt^ method. The primers used in this study are described in Additional file 1: Table S2.

### Differentiation assays in vitro

The targeted differentiation assays of neurons, skeletal muscles, and hepatocytes in vitro were carried out with a commercially available Quick-Trilineage™ Differentiation kit (EXGS-Q3D, Elixirgen Scientific, Baltimore, MD, USA) following the rapid differentiation protocols of neurons, skeletal muscles, and hepatocytes by mRNA encoding [31–33]. Undifferentiated hiPSCs after prolonged expansion were transferred to 24-well plates (CORNING, Corning, NY, USA) precoated with iMatrix-511 and maintained at the required chemical and physical conditions for a week as described in the protocols, and media were changed every day. Immunostaining was performed on day 7 with lineage-specific markers for neurons, skeletal muscles, and hepatocytes.

### Teratoma formation in vivo and histochemical assay

Animal experiments were carried out for the teratoma generation assay, following the Osaka University guidelines. 253G1 hiPSCs (0.5–1 × 10^7^) suspended in 100 µL of DMEM/F12-Matrigel (10%) were injected subcutaneously into 6–8-week-old female severe combined immunodeficiency (SCID) mice (C.B-17/Icr-scid/scidJcl, CLEA, Tokyo, Japan). After 8–10 weeks, the formed teratomas were fixed with formaldehyde solution and embedded in paraffin blocks for routine sectioning and staining with hematoxylin and eosin (HE). A CKX41 microscope (Olympus) was used for histological assessment of teratoma sections.

### Karyotyping

Karyotype analysis was performed on G-banded metaphase cells by Nihon Gene Research Laboratories, Inc. (Sendai, Japan). Twenty cells were analyzed for each sample.

### Statistical analysis

All quantitative data are presented as mean ± standard deviation of the mean (mean ± SD). The differences were analyzed using independent one-tailed Student’s t test or one-way ANOVA followed by the Tukey’s multiple comparison post hoc tests with significant differences defined as *p < 0.05, ** < 0.01, and *** < 0.001. The data shown are representative of three independent experiments.

## Results

### The developed serum- and xeno-free ON2 is capable of combination with various substrates

To validate the feasibility of ON2 for long-term feeder-free propagation of hiPSCs, we first maintained a 253G1 hiPSC line in ON2 on different matrices, including the iMatrix-511 and GNF. Though both matrices fit for single-cell passage that is beneficial for quality control during hiPSC long-term expansion, enzyme-free dissociation buffer (ethylenediaminetetraacetic acid, EDTA/PBS-based buffer) was especially applied for GNF, which greatly reduces cell damage induced by the enzymatic dissociation buffer and improves quality control (Additional file 2: Fig. S1) [21, 28]. As a positive medium control, we chose xenogeneic mTeSR1 as neither E8 nor AK02N, two common xeno-free media, could support the survival of hiPSCs on GNF, even in the presence of 10 µM Rho-associated protein kinase inhibitor (ROCKi) Y27632, which is known to prevent cell apoptosis induced by dissociation (Additional file 2: Fig. S2a).

**Fig. 1:**
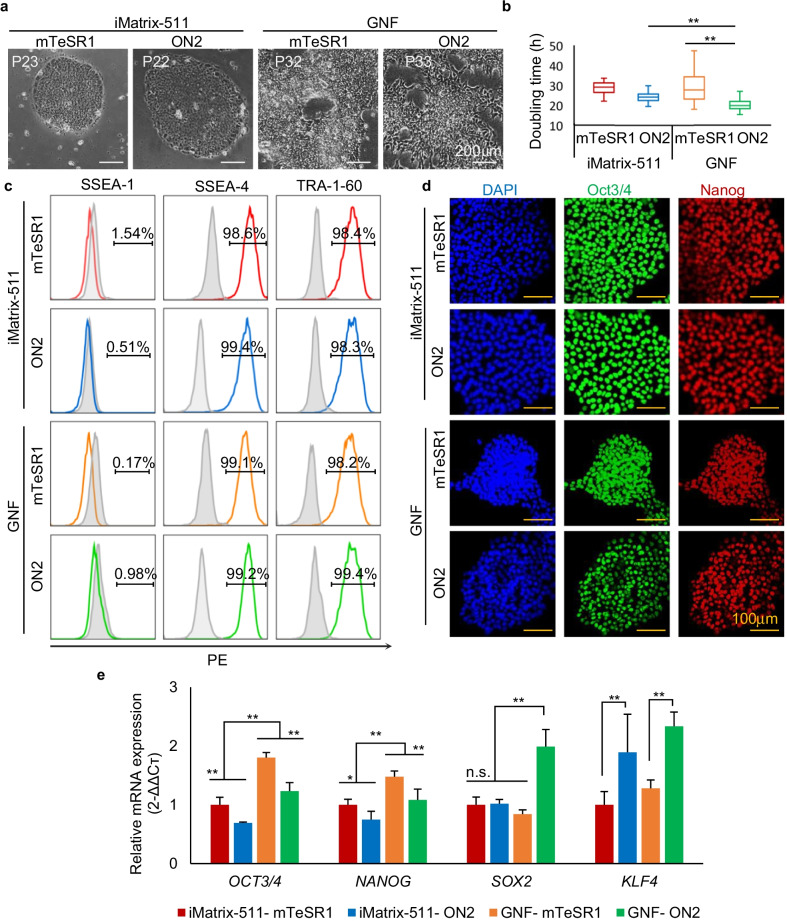
hiPSCs pluripotency after long-term expansion in ON2 medium on iMatrix-511 or GNF. **a** 253G1 hiPSC clone morphology on iMatrix-511 and GNF substrates over 20 passages with ON2. mTeSR1 was used as the positive control medium. GNF: gelatin nanofibrous. Scale bar, 200 µm. **b** Comparison of doubling times between 253G1 hiPSCs grown on iMatrix-511 and GNF, respectively (passage number = 20–33, **p < 0.01, data are mean ± SD). **c** Flow cytometric analysis of SSEA4, TRA-1-60, and SSEA1. Light-gray solid histograms show the isotype control populations, and color hollow histograms show the stained populations, respectively. The percentages of marker-positive cells are presented in each graph. Cell events were normalized to the mode. **d** Immunofluorescence images of hiPSC colonies stained for pluripotent markers Oct3/4 (green) and Nanog (red). Nuclei were stained with DAPI dye (blue). Scale bar, 100 µm. qRT-PCR was carried out to compare the expression levels of pluripotency genes *OCT4, NANOG, SOX2, and KLF4* in 253G1 hiPSCs cultured on iMatrix-511 and GNF with ON2 or mTeSR1. (*n* = 6 biological independent samples; Data are mean ± SD, *p < 0.05, **p < 0.01)

ON2 medium was sufficient for 253G1 hiPSC maintenance and long-term expansion on different matrices. After more than 20 passages, 253G1 hiPSCs in both ON2 medium and control medium mTeSR1 showed clear and compact clones on both substrates (Fig. 1a). As indicated by their respective average doubling time, when grown on iMatrix-511 in ON2, 253G1 hiPSCs were more proliferative and stable than those in the control (doubling time was 23.96 ± 2.44 h and 28.77 ± 3.09 h for ON2 and mTeSR1, respectively). The proliferation rate was further amplified in the media that contained GNF as the substrate (doubling time was 20.50 ± 3.77 h and 31.19 ± 12.64 h for ON2 and mTeSR1, respectively) (Fig. 1b). This indicated that cell proliferation could be robustly enhanced by both ON2 and GNF.

To evaluate the pluripotency of the grown cells, flow cytometry (FACS) assay, immunofluorescence (IF) staining, and quantitative real-time polymerase chain reaction (qRT-PCR) analysis were conducted to assess the expression level of pluripotent markers for hiPSCs. FACS assay showed that most 253G1 hiPSCs were positive for the cell surface markers of pluripotent cells, SSEA-4 and TRA-1-60, after long-term culture (Fig. 1c). IF staining of pluripotent cell markers indicated that most of the cells expressed Oct3/4 and Nanog after multiple passages (Fig. 1d). Although FACS and IF staining showed no distinct differences between different matrices, qRT-PCR indicated that the gene expression levels of *OCT3/4, NANOG, SOX2*, and *KLF4* were quite different in the cells grown on GNF and iMatrix-511 (Fig. 1e). When grown on iMatrix-511, cells in ON2 demonstrated lower expression of *OCT4* and *NANOG*, but significantly higher levels of *KLF4* than those in mTeSR1. A similar gene expression pattern was observed in 253G1 cells cultivated on GNFs. However, GNF showed an elevation of almost all genes compared to iMatrix-511, indicating that not only the culture medium, but also the coating substrates affect the characteristics of hiPSCs.

**Fig. 2.**
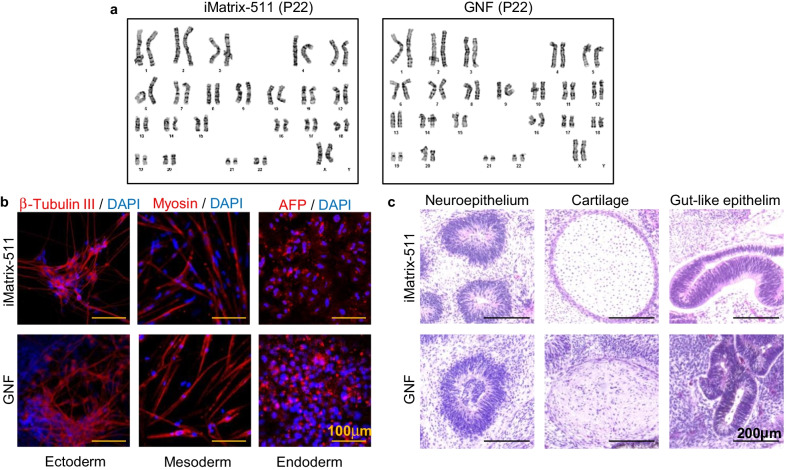
253G1 hiPSCs on iMatrix-511 and GNF with ON2 medium maintained normal karyotype and differentiation ability. **a** Normal karyotypes of 253G1 hiPSCs maintained on both iMatrix-511 and GNF over 20 passages with ON2 were identified by G-band karyotype analysis (46 XX). **b** All three germ layer differentiation in vitro of 253G1 hiPSCs occurred after expansion on iMatrix-511 or GNF with ON2. Ectodermal, mesodermal, and endodermal cell types were revealed by specific markers: β-tubulin III, myosin, and AFP, respectively, by immunocytochemistry. Scale bar, 100 µm. **c** Histological analysis of teratomas generated in SCID mice from 253G1 iPSCs after ON2 culture on iMatrix-511 or GNF, respectively. Neuro-epithelium, cartilage, and gut-like epithelium were visualized by hematoxylin and eosin staining. Scale bar, 200 µm

Furthermore, 253G1 hiPSCs grown on GNF maintained a normal karyotype (46 XX) without chromosomal abnormalities, similar to those on iMarix-511, which was confirmed by G-banding karyotype analysis (Fig. 2a). This indicated the ability of xeno-free ON2 to maintain the genomic stability of hiPSCs after long-term expansion.

To examine whether hiPSCs retain their potential to differentiate into various somatic cells, directed differentiation assays in vitro and teratoma formation assays in vivo were conducted after prolonged passaging in ON2. After 20 passages, 253G1 hiPSCs were collected from iMatrix-511 and GNF and subsequently induced with a lineage-specific kit of ectoderm, mesoderm, and endoderm. As expected, the 253G1 hiPSCs were positive for β-tubulin (ectoderm, neuron marker), myosin (mesoderm, skeletal muscle marker), and alpha-fetoprotein (endoderm, hepatocyte marker) (Fig. 2b), indicating that hiPSCs grown on both matrices can be induced into three germ layers in vitro after propagation in ON2. Furthermore, teratoma formation was induced by subcutaneous injection of 0.5–1 × 10^7^ hiPSCs in 8-week-old female SCID (C.B-17/Icr-scid/scidJcl, CLEA, Tokyo, Japan) mice. Eight to 10 weeks after injection, teratomas were obtained from all cell groups, and histological analysis was carried out. Neuro-epithelium, cartilage, and gut-like epithelium could be seen in the different groups, via HE staining (Fig. 2c and Additional file 2: Fig. S2b). These results demonstrated that both matrices with ON2 maintained the differentiation potential of hiPSCs and that ON2 was as capable of sustaining hiPSCs. Although GNF showed a slightly superior support for hiPSCs, more studies are required before commercialization.

We also evaluated two other common commercial substrates, human plasma FN and truncated recombinant human VN, in combination with ON2, for hiPSCs. It has been reported that both FN and VN allow single-cell passaging; however, in our study, we observed that both FN and VN preferred cluster passaging over single-cell passaging because single cells had poorer survival than clusters on both matrices. For FN, a higher coating concentration was needed than VN, as thin FN seemed insufficient to support cell growth, according to preliminary experiments (Additional file 2: Fig. S3a, b). Thus, we applied 2.0 µg/cm^2^ FN and 0.5 µg/cm^2^ VN in combination with ON2, for the prolonged expansion of 253G1 hiPSCs. After more than 10 passages, 253G1 cells displayed accurate morphology without distinct differentiation and were positive for all detected pluripotent markers, as verified by IF, FACS, and qRT-PCR analysis (Additional file 2: Fig. S4a–d). In short, we demonstrated the compatibility of ON2 with various substrates for the expansion of hiPSCs. Considering aspects such as cost, convenience, and reliability, we chose iMatrix-511 as the optimal substrate for ON2 for the following experiments.

### Comparison of different xeno-free culture mediums for hiPSCs

**Fig. 3:**
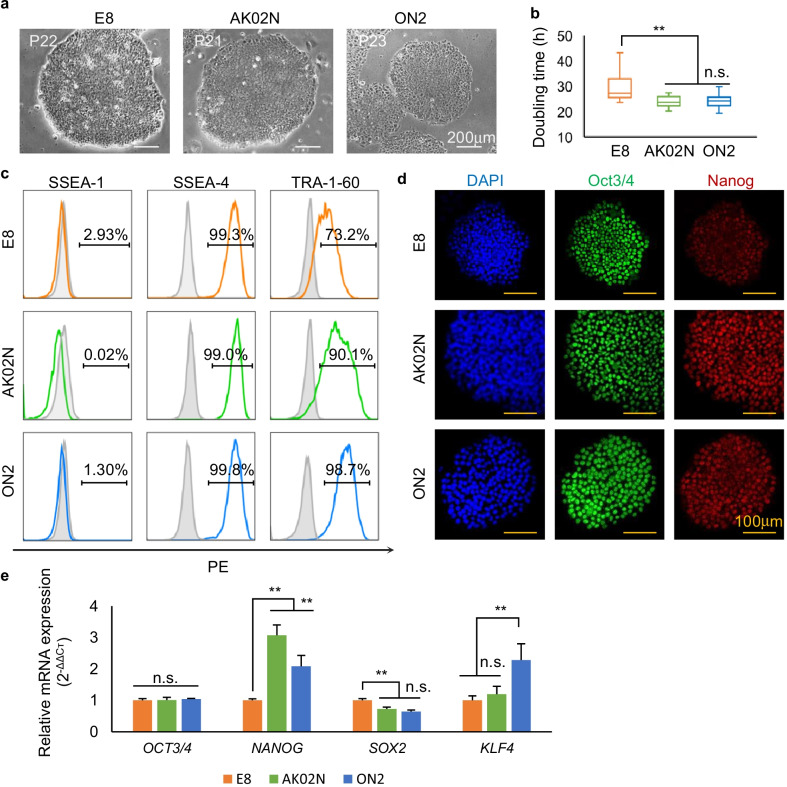
ON2 showed equal or superior efficiency compared to common xeno-free culture media. **a** 253G1 hiPSC clone morphology on iMatrix-511 with E8, AK02N, or ON2, respectively, over 20 passages. Scale bar, 200 µm. **b** Doubling time comparison of 253G1 hiPSCs maintained in different xeno-free culture media: E8, AK02N, or ON2. (Passage number = 20, **p < 0.01, n.s., no significant difference; data are mean ± SD). **c** Flow cytometric analysis of SSEA4, TRA-1-60, and SSEA1. Gray solid histograms show the isotype control populations, and color hollow histograms show the stained populations. The percentages of marker-positive cells are presented in each graph. Cell events were normalized to the mode. **d** Immunofluorescence staining of pluripotency markers in hiPSCs: Oct3/4 (green); Nanog (red). Nuclei were stained with DAPI dye (blue). Scale bar, 100 µm. **e** qRT-PCR analysis of pluripotent gene expression levels of *OCT4, NANOG, SOX2, and KLF4* in 253G1 hiPSCs in three different xeno-free culture media. (*n* = 6 independent samples, and data are mean ± SD, *p < 0.05, ** or ^##^*p* < 0.01, *n.s.* no significant difference)

As both E8 and AK02N are commercially available xeno-free media for hiPSCs, we compared the efficiency of using ON2 with them. For these experiments, iMatrix-511 was used as the common coating matrix because neither E8 nor AK02N could sustain hiPSCs on GNF (Additional file 2: Fig. S2a), and 253G1 hiPSCs grown on iMatrix-511 were maintained in ON2, E8, or AK02N for at least 20 sequential passages. Typically, compact clone morphology was observed throughout the whole expansion (Fig. 3a). Cell proliferation was evaluated by average doubling time (29.58 ± 5.21 h for E8, 24.50 ± 3.95 h for AK02N, and 23.96 ± 2.38 h for ON2, respectively). hiPSCs in ON2 or AK02N were significantly more proliferative and stable than those in E8, as verified by their shorter doubling time (Fig. 3b). The pluripotency of expanded 253G1 hiPSCs was evaluated by analyzing the expression levels of pluripotent markers. FACS analysis indicated that > 98% of 253G1 cells grown in ON2 were positive for SSEA4 and TRA-1-60, while cells in AK02N and E8 showed lower positivity for TRA-1-60 (90.1% for AK02 and 73.2% for E8), even though similar SSEA4 positive ratios (> 99%) were still observed (Fig. 3c). In addition, although 253G1 hiPSCs in each medium expressed the pluripotent markers Oct3/4 and Nanog, the fluorescence intensity of Nanog was weaker in E8, as revealed by IF staining (Fig. 3d). qRT-PCR analysis also confirmed similar *OCT3/4* expression but lower *NANOG* expression, as well as a slightly higher *SOX2* expression in cells maintained in E8 than in ON2 or AK02N (Fig. 3e). In addition, the expression level of *KLF4* was significantly higher when cells were grown in ON2 than in the other media. Even though there were greater variations in the gene expression levels of undifferentiated markers, we believe that the xeno-free ON2 showed comparable capability to AK02N and was superior to E8 in maintaining hiPSCs in terms of both propagation and pluripotency.

### The long-term expansion of multiple hiPSC lines in ON2

**Fig. 4:**
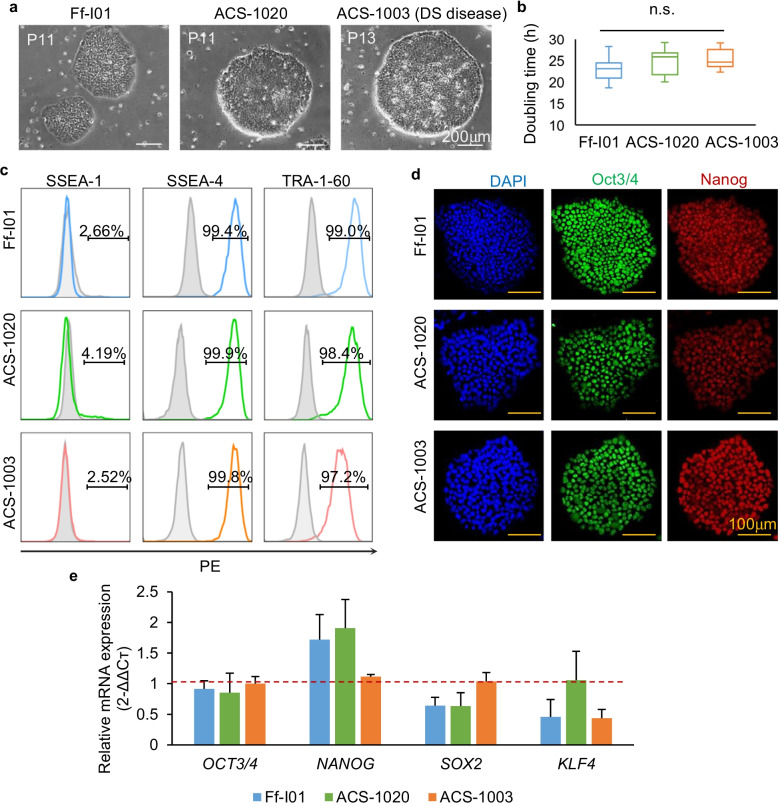
ON2 medium sustains the prolonged expansion of different hiPSC lines. **a** Ff-l01, ACS-1020, and ACS-1003 hiPSC clones after long-term expansion of iMatrix-511 in the ON2 medium. Scale bar, 200 µm. **b** Comparison of doubling times for Ff-l01, ACS-1020, and ACS-1003 hiPSCs cultured in ON2 (passage number = 12–15, *n.s.* no significant difference; data are mean ± SD) (Ff-l01: 23.09 ± 2.88; ACS-1020: 24.78 ± 3.02; ACS-1003: 25.28 ± 2.21. **c** FACS analysis of pluripotent marker-positive cells. Light-gray solid histograms show the isotype control populations, and color hollow histograms show the stained populations, respectively. The percentages of maker-positive cells are presented in each graph. Cell events were normalized to mode. **d** Immunofluorescence staining of pluripotency markers in hiPSCs. Green: Oct3/4; red: Nanog; blue: DAPI. Scale bar, 100 µm. **e** qRT-PCR analysis of pluripotent gene expression levels of *OCT4, NANOG, SOX2, and KLF4* in three hiPSCs sustained in ON2. Fold expression was compared to that in 253G1 hiPSC cell lines in ON2. (*n* = 3 biological independent sample, *n.s.* no significant difference; data are mean ± SD)

We assessed the capability of ON2 to support the propagation of multiple cell lines, including a healthy hiPSC line (ACS-1020), an HLA-homo hiPSC line (Ff-l01), and a DS-derived hiPSC line (ASC-1003). ON2 was effective in maintaining the survival, growth, and stemness of all cultured hiPSC lines on the iMatrix-511 substrate. Through 10 passages in ON2, large, round clone morphology without spontaneous differentiation was observed, and no differences were found in terms of proliferation in all tested hiPSC lines (Fig. 4a, b, doubling times were 23.09 ± 2.88 h for Ff-l01, 24.78 ± 3.02 h for ACS-1020, and 25.28 ± 2.21 h for ACS-1003), similar to 253G1 hiPSCs (Fig. 1a). We also evaluated the expression levels of pluripotent markers for all propagated hiPSCs in ON2. ACS-1020, Ff-l01, and ACS-1003 hiPSCs grown in ON2 were positive for all undifferentiated markers, SSEA-4, TRA-1-60, Oct3/4, and Nanog, as verified by FACS analysis and IF staining (Fig. 4c, d). Although the relative gene expression profile was slightly turbulent, as revealed by qRT-PCR analysis, an insignificant difference was found among these hiPSC lines (Fig. 4e).

As a positive control, E8 medium was applied to sustain Ff-l01 hiPSCs, a line used in clinical research (Additional file 2: Fig. S6). Over 15 passages, the proliferation and pluripotency of Ff-l01 were evaluated. Normal round clonal morphology without spontaneous differentiation was observed by light microscope (Additional file 2: Fig. S6a), similar to that observed in ON2 (Fig. 4a). However, lower proliferation of Ff-l01 in E8 was observed than in ON2, which was confirmed by their doubling times: 27.0 ± 4.07 h in E8 and 23.09 ± 2.88 h in ON2, respectively (Additional file 2: Fig. S6b). For pluripotency, Ff-l01 hiPSCs in E8 were less positive to TRA-1-60 comparing to those in ON2, though no significant difference was found in other detected markers (Additional file 2: Fig. S6c, d; Fig. 4). These results indicated that the xeno-free ON2 culture medium could universally sustain the survival, pluripotency, and robust propagation of hiPSCs during expansion, suggesting the potential of ON2 for further clinical applications such as disease modeling, drug development, and cell therapy.

## Discussion

We developed a cost-effective and xeno-free medium, ON2, which was compatible with various substrates including iMatrix-511, FN, VN, and GNF, and which sustained the survival, self-renewal, and pluripotency of hiPSCs, even after prolonged expansion. When compared to other common commercial xeno-free media, ON2 effectively promoted proliferation and maintained the pluripotency of cultured hiPSCs. Moreover, ON2 could sustain not only healthy donor-derived hiPSC lines including 253G1 and ACS-1020 but also the HLA-homo Ff-l01 and the DS patient-specific hiPSC line ACS-1003.

The establishment of hiPSCs in 2007 offered a clinically applicable and effective culture process for large-scale production. Substantial efforts have been made to improve the culture substrate, medium component, and passage method for this purpose [13, 34]. ECM mediates cell–matrix interactions by activating integrins located on the cell surface, promoting cell survival, self-renewal, and differentiation [35, 36]. So far, human recombinant ECM matrix substrates have been generated to replace feeder cell or Matrigel for clinically oriented hiPSC expansion. In the present study, we tested three commercialized matrices, FN, VN, and iMatrix-511, as well as a low-adhesive substrate, GNF, and all were suitable for the propagation of hESCs/hiPSCs. All applied matrices have been reported previously to be reliable and reproducible for hiPSC expansion [21, 27, 28, 37]. According to our results, all coatings, especially iMatrix-511 and GNF, successfully sustained the survival and growth of hiPSCs combined with ON2. After long-term expansion, no spontaneous differentiation was observed in the hiPSCs grown in these matrices. It has been reported that all tested matrices in this study allowed for single-cell passage [20, 28, 37]. However, our results showed that completely dissociated hiPSCs showed poor survival and adhesion on either FN or VN, even in the presence of ROCKi, indicating that FN and VN could only work with cluster expansion rather than single cells [20, 37]. The cluster passage method may introduce unpredictable proliferation of hiPSCs, and it is difficult to control the cluster quality during large-scale expansion. In addition, a higher coating concentration of FN (four times higher than that for VN and iMatrix-511) is required for cultured hiPSCs, which could lead to higher costs than those for other tested matrices [18].

Laminin is reported to be essential for early embryonic development and is widely distributed in various tissues. It has become popular as a natural scaffold for the derivation, expansion, and differentiation of hiPSCs, from laboratory research to clinical trials [9]. Laminin-511 is one of the most commonly used laminin isoforms for sustaining hESCs, and its developed recombinant E8 fragments, commercialized as iMatrix-511, showed high adhesion and support for hiPSCs compared to other ECM proteins combined with various xenogeneic or xeno-free media [18]. Similarly, in our work, we demonstrated that iMatrix-511 showed better cell adhesion and stability with ON2 than other tested commercial matrices [38]. In addition, the single-cell passage and enzyme-free dissociation method and lower ROCKi requirement enhanced the efficiency and stability of cell expansion [39].

GNF is a novel nanoscale coating material with a low-attachment surface and is competent for the expansion of hiPSCs, as shown in our previous studies. The low-adhesive profile of this substrate is capable of sorting subtypes within hiPSCs, including flat (monolayered) morphology and domed (multilayered) morphology with different pluripotency [21, 22]. In addition, enzyme-free single-cell passage with GNF significantly reduces the risk of cell damage and spontaneous differentiation. However, GNF could not adjust to common xeno-free media such as AK02 and E8. This could be because the low-attachment GNF needs more nutrients apart from albumin for the sustenance of hiPSCs grown on it. Based on this hypothesis, we developed a nutritious but xeno-free medium, ON2. With ON2, GNF maintained hiPSCs for over 30 passages without morphological and chromosomal abnormalities. More importantly, hiPSCs grown on GNF showed higher proliferation than those on iMatrix-511 in this study. The morphology of the colony also varied on different substrates, with many protruding regions forming on the edge, clinging tightly to the GNF substrate. The nanofibrous structure of GNF may contribute to the tissue-like formation of hiPSCs and may alter the growth pattern by unknown mechanisms [40]. It has also been reported that the low-attachment GNF surface may provide a low-cell ECM attachment but high cell–cell adhesion regulated by E-cadherin signaling, which benefits cell self-renewal [21, 41]. Although GNF showed advantages in the maintenance of hiPSCs, more evaluative work is needed before GNF can be commercially available.

Culture medium is another critical component of the culture system, as it provides various growth factors and an optimal required and suitable pH microenvironment for maintaining hiPSCs. In this study, apart from our ON2, we also applied three commercial media including xenogeneic mTeSR1 and xeno-free E8 and AK02N, all of which are commonly used media for hESC/hiPSCs in routine study. mTeSR1 is a complex medium containing several growth factors and signaling agonists that promote hESC growth by stimulating their self-renewal signaling pathway [24, 26]. Although it is xenogeneic, mTeSR1 has been widely applied to diverse culture systems from feeder to feeder-free systems for hESC/hiPSC propagation [15, 25]. In our study, mTeSR1 stably maintained the pluripotency and differentiation potential of hiPSCs on both iMatrix-511 and low-attached GNF (Fig. 1 and Additional file 2: Fig. S2b). Furthermore, mTeSR1 seems to enhance the expression level of *NANOG* and *OCT3/4* of grown hiPSCs, while ON2 improved *KLF4* expression specifically.

The simplest xeno-free E8 contains only four growth factors but no albumin, and it is sufficient for the maintenance of hiPSCs combined with different matrices such as VN and iMatrix-511. In our study, 253G1 hiPSCs grown in E8 showed lower proliferation and were prone to spontaneous differentiation over 20 passages (Additional file 2: Fig. S5a). Furthermore, chromosomal abnormality was detected by karyotype analysis (Additional file 2: Fig. S5b), but such morphological variation was not found in the Ff-l01 cells grown in E8 within 15 passages (Additional file 2: Fig. S6). The behavior of E8 may vary between cell lines. However, considering that cells in AK02N and ON2 did not have the above abnormality under the same conditions, we speculate that the lower pluripotency of cells in E8 was related to the lack of albumin, which was in both AK02N and ON2 (Additional file 1: Table S1). It has been reported that albumin can neutralize acidosis, which is responsible for cell differentiation, so we speculated that albumin is essential for the long-term expansion of undifferentiated cells [42]. In this study, the albumin-contained AK02N is efficient and capable for the stable expansion of hiPSCs maintained on iMatrix-511 and no spontaneous differentiation was discovered after propagation (Fig. 3). While comparing to ON2, AK02N displayed less positive to some pluripotent markers such as TRA-1-60, and moreover, it is not sufficient for the cells grown on the low-attached GNF substrate (Additional file 2: Fig. S2a).

The capability for culture medium to maintain pluripotency and robustness of cultured cells is of great importance. It has been reported that the cell cycle of hESCs / hiPSCs could be reduced to 16–20 h under optimized culture condition, which is very close to the hESCs in vivo (16–18 h) [43–47]. In addition, prolonged cell cycle may lead to spontaneous differentiation [48, 49]. In the present study, higher proliferative effect of ON2 on hiPSCs (similar to AK02N, but much robust than E8) was verified by the doubling times (around 20–24 h). Moreover, the pluripotent stem cells maintained in ON2 demonstrated high pluripotent potential and adaptable adhesive capability to various extracellular matrix, which may be related to the enhanced *KLF4* expression level. Versatile transcriptional factor *KLF4* has been reported to directly induce E-cadherin expression in mesenchymal–epithelial transition and promote the metabolic shift into anaerobic glycolysis during somatic cell reprogramming, both are beneficial for robust proliferation of pluripotent stem cells [23, 50, 51]. We have previously reported that the upregulation of *KLF4* could lead to cell cycle acceleration, and such variation is accompanied by changeable cell–matrix adhesion [23], which is consistent with the finding in the present work.

Growth factor-based medium is a major focus of hiPSC research, and most popular commercial media, such as mTeSR1 and E8, contain the two factors FGF2 and TGFβ, which drive hPSC pluripotency by activating PI3K/AKT/mTOR pathways and TGFb signaling pathways, respectively, but the high cost of these factors remains a challenge (Additional file 1: Table S1) [24, 26, 27]. We have made some effort toward developing components to control the cost of ON2. On the one hand, we removed TGFβ and reduced the dose of FGF2, which is a thermally unstable growth factor that is usually included in media at a higher dose than is needed. Instead, we supplemented the medium with the cost-effective activin A, which functions similar to TGFβ, LIF that seems to be necessary for more pluripotent state cells, and albumin that acts as a multifaceted antioxidant [42, 46, 52–56]. Although ON2 is more cost-effective than commercial media, it is still dependent on the thermally unstable factor FGF2. Recently, a negligible cost and chemically defined medium for hiPSCs was developed (B8). Compared to E8 and ON2, B8 includes fewer growth factors, including thermostable but more potent FGF2 variants, to reduce the cost [57]. The combination of E8 and Matrigel will not be suitable for clinical applications. AKIT, a growth factor-free medium, is stable for hiPSC culture and is more cost-effective because the expensive and unstable proteins that are included in most culture media have been removed [58]. AKIT medium sustained hiPSCs at the expense of decreased proliferation and poor single-cell survival, claimed by the authors. Although these media are not perfect, they offer novel options to improve ON2 so that it can be more suitable for large-scale expansion of hiPSCs in the future. Besides, further research is needed to meet the quality and safety standards of Good Manufacturing Practices.

## Conclusion

In summary, we have developed a complete xeno-free culture medium for hiPSC expansion with improved efficiency, repeatability, and stability in pluripotency and differentiation potential. ON2 medium is compatible with various matrices, including fibronectin, vitronectin, laminin, and gelatin nanofibers for the sustenance of hiPSCs. We compared the efficiency of ON2 with that of commercially used media and found that ON2 showed comparable ability to support hiPSCs. Moreover, we applied ON2 to the cultivation of not only normal hiPSC lines, including 253G1 and ACS-1020, but also HLA-homo Ff-l01 and Down's syndrome patient-specific hiPSC line ACS-1003. All tested hiPSCs could robustly expand over at least 10 passages in ON2 with clear clone morphology and high expression of pluripotent genes. Our results indicate that the developed xeno-free culture medium ON2 is viable and reliable and provides a powerful tool for both basic research and clinical applications of hiPSCs.

## Supplementary Information

**Additional file 1: Table S1.** Media components of TeSR, E8 and ON2. **Table S2.** Primers used in qRT-PCR analysis

**Additional file 2: Fig. S1.** 253G1 maintained on GNF in ON2 or mTeSR1. Most of seeded single cells attached to GNF within 2 h and protruding regions were formed on the edge the clones, clinging tightly to the GNF substrate. Scale bar, 200 µm. **Fig. S2.** Commercial xeno-free media could not support hiPSCs on GNF. (a) Neither AK02N nor E8 could maintain the hiPSCs on GNF. Most the cells apoptosis induced from P2 even with 10 µM Rock inhibitor. On the contrary, ON2 shares equal capability as mTeSR1 for maintaining survival and adhesion of cultured hiPSCs. Scale bar, 200 µm. (b) Teratomas generated from 253G1 after maintained on GNF with mTeSR1 for over P20. Scale bar, 100 µm. **Fig. S3.** Optimization of coating conditions of FN and VN. (a) 253G1 cultured on FN with a coating concentration of 0.5 g/ml, 1.0 g/ml or 2.0 g/ml. Scale bar, 200 µm. (b) 253G1 maintained on VN with a coating concentration of 0.5 g/ml. Scale bar, 200 µm. **Fig. S4.** Characterization of 253G1 hiPSCs cultured on FN and VN in ON2. (a) 253G1 colony morphology on FN and VN at P10. Scale bar, 200 µm. (b) qRT–PCR gene expression analysis of 253G1 hiPSCs grown on FN and VN in ON2. Fold expression is compared to cells cultured on iMatrix-511 in ON2 (n = 3 biological replicates, data are mean ± SD). (c) FACS analysis of pluripotency marker-positive cells. light-gray solid histograms show the isotype control populations, and green and blue hollow histograms show the stained populations on FN and VN, respectively. The percentage of maker-positive cells is presented on each graph. Cell events were normalized to mode. (d) Immunostaining of pluripotency markers. Green: Oct3/4; Red: Nanog; Blue: DAPI. Scale bar, 100 µm. **Fig. S5.** 253G1 cells cultured in E8 showed abnormalities after long-term expansion. (a) Spontaneous differentiation was found in 253G1 hiPSCs maintained on iMatrix-511 with E8 medium over P20, verified by immunostaining of hiPSCs for pluripotent markers: Oct3/4 (green); Nanog (red). Nuclei were stained with DAPI (blue). Scale bar, 100 µm. (b) Karyotype abnormality of 253G1 hiPSCs on iMatrix-511 with E8 at P22, identified by G-band karyotype analysis. **Fig. S6.** Characterization of HLA-homo Ff-l01 hiPSCs cultured in E8. (a) Ff-l01 colonel morphology in E8 at P14. Scale bar, 200 µm. (b) Comparison of doubling times for Ff-l01 grown in E8 with those in ON2 (passage number = 15, **p < 0.01, data are mean ± SD). (c) Immunostaining of pluripotency markers. Green: Oct3/4; Red: Nanog; Nuclei were stained with DAPI (blue). Scale bar, 200 µm. (d) FACS analysis of pluripotency marker-positive cells. light-gray solid histograms show the isotype control populations, and blue hollow histograms show the stained Ff-l01 populations in E8. The percentage of maker-positive cells is presented on each graph. Cell events were normalized to mode.

## Data Availability

The original data are available from the corresponding author upon reasonable request.

## Abbreviations

AK02N: StemFit AK02N medium
bFGF: Basic fibroblast growth factor
DS: Down syndrome
E8: Essential 8 medium
ECM: Extracellular matrix
EDTA: Ethylenediaminetetraacetic acid
FACS: Fluorescence-activated cell sorter or flow cytometry
FBS: Fetal bovine serum
FN: Fibronectin
GNF: Gelatin nanofiber
HE: Hematoxylin and eosin staining
hESC: Human embryonic stem cells
hiPSC: Human-induced pluripotent stem cells
HLA: Human leukocyte antigen
IF: Immunofluorescence
LIF: Leukemia inhibitory factor
LM511-E8: Laminin-511 E8-fragment
MEF: Mouse embryonic fibroblasts
ON2: ON2/AscleStem PSC medium
qRT-PCR: Quantitative real-time polymerase chain reaction
ROCKi: Rho-associated protein kinase inhibitor
SCID: Severe combined immunodeficiency
TGFβ: Transforming growth factor beta
VN: Vitronectin
