# Supplementary material for: Development and evaluation of a novel xeno-free culture medium for human-induced pluripotent stem cells
Source: Stem Cell Res Ther. 2022 Jun 3;13:223. doi: 10.1186/s13287-022-02879-z (PMC9166585; doi:10.1186/s13287-022-02879-z)
Supplement: Supplementary file 1 — Additional file 1: Table S1. Media components of TeSR, E8 and ON2. Table S2. Primers used in qRT-PCR analysis [file 13287_2022_2879_MOESM1_ESM.pdf]

Table S1 Media components of TeSR, E8 and ON2

Components	TeSR[1]	E8[2]	ON2	Storage
DMEM/F12				Basal medium (At 4°C)
$NaHCO_3$				
L-Ascorbic Acid				
Selenium				
Insulin				
Transferrin				
FGF2				Supplement (Below -20°C)
TGFβ1				
Albumin				
Pipecolic acid				
LiCl				
GABA				
Glutathione				
L-Glutamine				
Defined lipids				
Thiamine				
Trace elements B				
Trace elements C				
β-mercaptoethanol				
NODAL				
Activin A [3-5]				
LIF [6-8]				
Chondroitin sulfate [9]				

Note 1: In E8 media, NODAL and TGFβ1 are interchangeable in maintaining ES and iPS cells.

Note 2: It is recommended to all three media that before use, thaw and mix supplement to basal medium to make into complete medium, which should be stored at 4°C temporary and used within two weeks.

[1] Ludwig, T.E., et al., Derivation of human embryonic stem cells in defined conditions. Nat Biotechnol, 513 2006. 24(2): p. 185-7

[2] Chen, G., et al., Chemically defined conditions for human iPSC derivation and culture. Nat Methods, 517 2011. 8(5): p. 424-9

[3] Vallier, L., et al., Activin/Nodal and FGF pathways cooperate to maintain pluripotency of human embryonic stem cells. J Cell Sci, 118 2005. (Pt 19):4495-509.

Pauklin, S. and L. Vallier, Activin/Nodal signalling in stem cells. Development, 2015. 142(4): p. 607-19.

[4] Osnato, A., et al., TGFbeta signalling is required to maintain pluripotency of human naive pluripotent stem cells. Elife, 2021. 10.

[5] Vallier, L., M. Alexander, and R.A. Pedersen, Activin/Nodal and FGF pathways cooperate to maintain pluripotency of human embryonic stem cells. Journal of Cell Science, 2005. 118(19): p. 4495-4509.
[6] Amit, M., et al., Feeder Layer- and Serum-Free Culture of Human Embryonic Stem Cells. Biol Reprod, 2004. 70(3): p. 837-45
[7] Gafni, O., et al., Derivation of novel human ground state naive pluripotent stem cells. Nature, 2013. 504(7479): p. 282-286.
[8] Ware Carol, B., et al., Derivation of naïve human embryonic stem cells. Proceedings of the National Academy of Sciences, 2014. 111(12): p. 4484-4489.
[9] Ida, M., et al., Identification and functions of chondroitin sulfate in the milieu of neural stem cells. J Biol Chem, 2006. 281(9): p. 5982-91

Table S2 Primers used in qRT-PCR analysis

Primer	Sequence
GAPDH	F: ACCACAGTCCATGCCATCAC<br>R: TCCACCACCCTGTTGCTGTA
OCT3/4	F: GACAGGGGGAGGGGAGGAGCTAGG<br>R: CTTCCCTCCAACCAGTTGCCCCAAAC
NANOG	F: CAGCCCCGATTCTTCCACCAGTCCC<br>R: CGGAAGATTCCCAGTCGGGTTCACC
SOX2	F: GGGAAATGGGAGGGGTGCAAAAGAC<br>R: TTGCGTGAGTGTGGATTGGTG
KLF4	F: CGCCTCCTGCTTGATCTTGGG<br>R: GACCCATCCTCCGGAGTCAGT
