# Supplementary material for: Development and evaluation of a novel xeno-free culture medium for human-induced pluripotent stem cells
Source: Stem Cell Res Ther. 2022 Jun 3;13:223. doi: 10.1186/s13287-022-02879-z (PMC9166585; doi:10.1186/s13287-022-02879-z)
Supplement: Supplementary file 2 — Additional file 2: Fig. S1. 253G1 maintained on GNF in ON2 or mTeSR1. Most of seeded single cells attached to GNF within 2 h and protruding regions were formed on the edge the clones, clinging tightly to the GNF substrate. Scale bar, 200 µm. Fig. S2. Commercial xeno-free media could not support hiPSCs on GNF. (a) Neither AK02N nor E8 could maintain the hiPSCs on GNF. Most the cells apoptosis induced from P2 even with 10 µM Rock inhibitor. On the contrary, ON2 shares equal capability as mTeSR1 for maintaining survival and adhesion of cultured hiPSCs. Scale bar, 200 µm. (b) Teratomas generated from 253G1 after maintained on GNF with mTeSR1 for over P20. Scale bar, 100 µm. Fig. S3. Optimization of coating conditions of FN and VN. (a) 253G1 cultured on FN with a coating concentration of 0.5 g/ml, 1.0 g/ml or 2.0 g/ml. Scale bar, 200 µm. (b) 253G1 maintained on VN with a coating concentration of 0.5 g/ml. Scale bar, 200 µm. Fig. S4. Characterization of 253G1 hiPSCs cultured on FN and VN in ON2. (a) 253G1 colony morphology on FN and VN at P10. Scale bar, 200 µm. (b) qRT–PCR gene expression analysis of 253G1 hiPSCs grown on FN and VN in ON2. Fold expression is compared to cells cultured on iMatrix-511 in ON2 (n = 3 biological replicates, data are mean ± SD). (c) FACS analysis of pluripotency marker-positive cells. light-gray solid histograms show the isotype control populations, and green and blue hollow histograms show the stained populations on FN and VN, respectively. The percentage of maker-positive cells is presented on each graph. Cell events were normalized to mode. (d) Immunostaining of pluripotency markers. Green: Oct3/4; Red: Nanog; Blue: DAPI. Scale bar, 100 µm. Fig. S5. 253G1 cells cultured in E8 showed abnormalities after long-term expansion. (a) Spontaneous differentiation was found in 253G1 hiPSCs maintained on iMatrix-511 with E8 medium over P20, verified by immunostaining of hiPSCs for pluripotent markers: Oct3/4 (green); Nanog (red). Nuclei were stained w [file 13287_2022_2879_MOESM2_ESM.pdf]

Figure S1

b

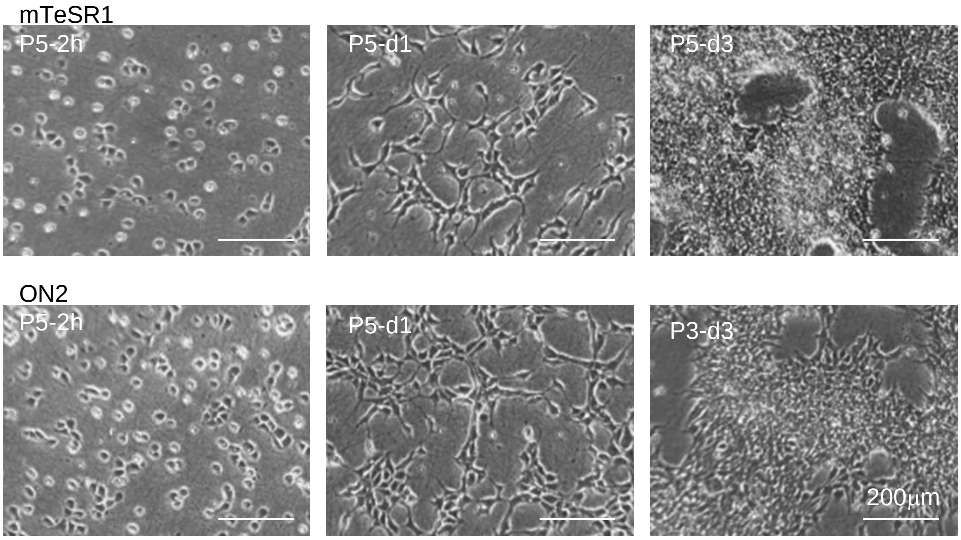

Figure S2

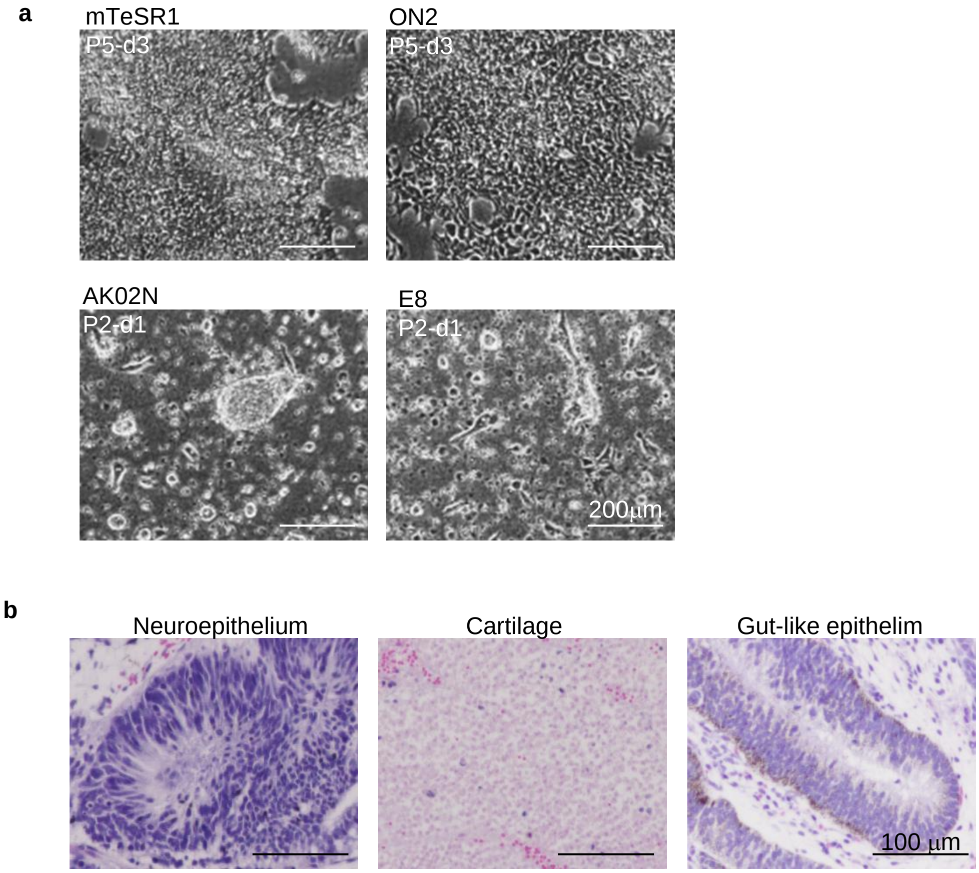

a
mTeSR1
P5-d3
ON2
P5-d3
AK02N
P2-d1
E8
P2-d1
200μm
b
Neuroepithelium
Cartilage
Gut-like epithelim
100 μm

Figure S3

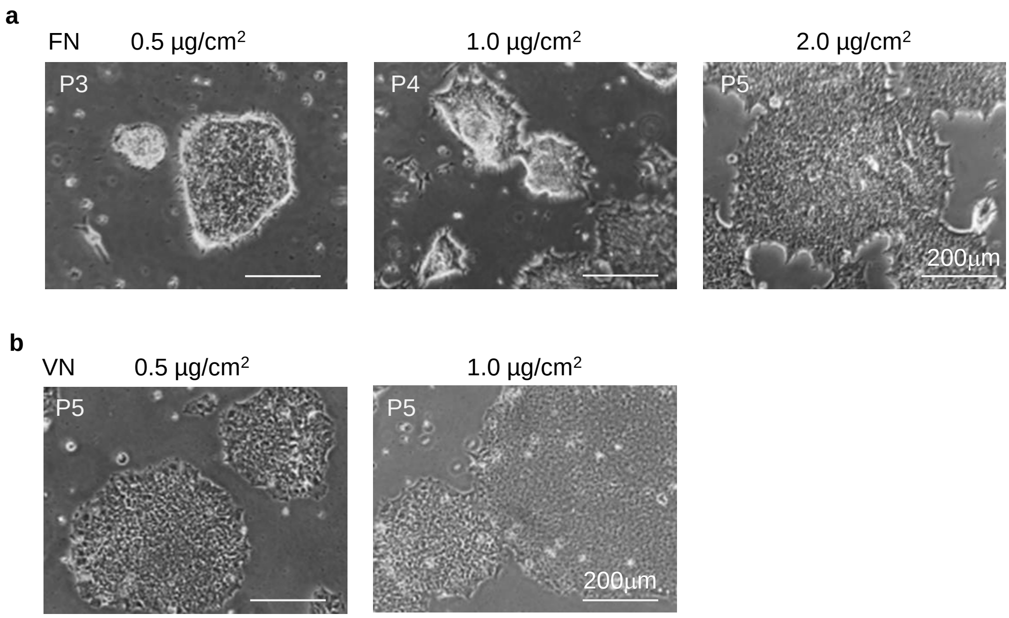
a
FN 0.5 µg/cm2
1.0 µg/cm2
2.0 µg/cm2
P3
P4
P5
200µm
b
VN 0.5 µg/cm2
1.0 µg/cm2
P5
P5
200µm

Figure S4

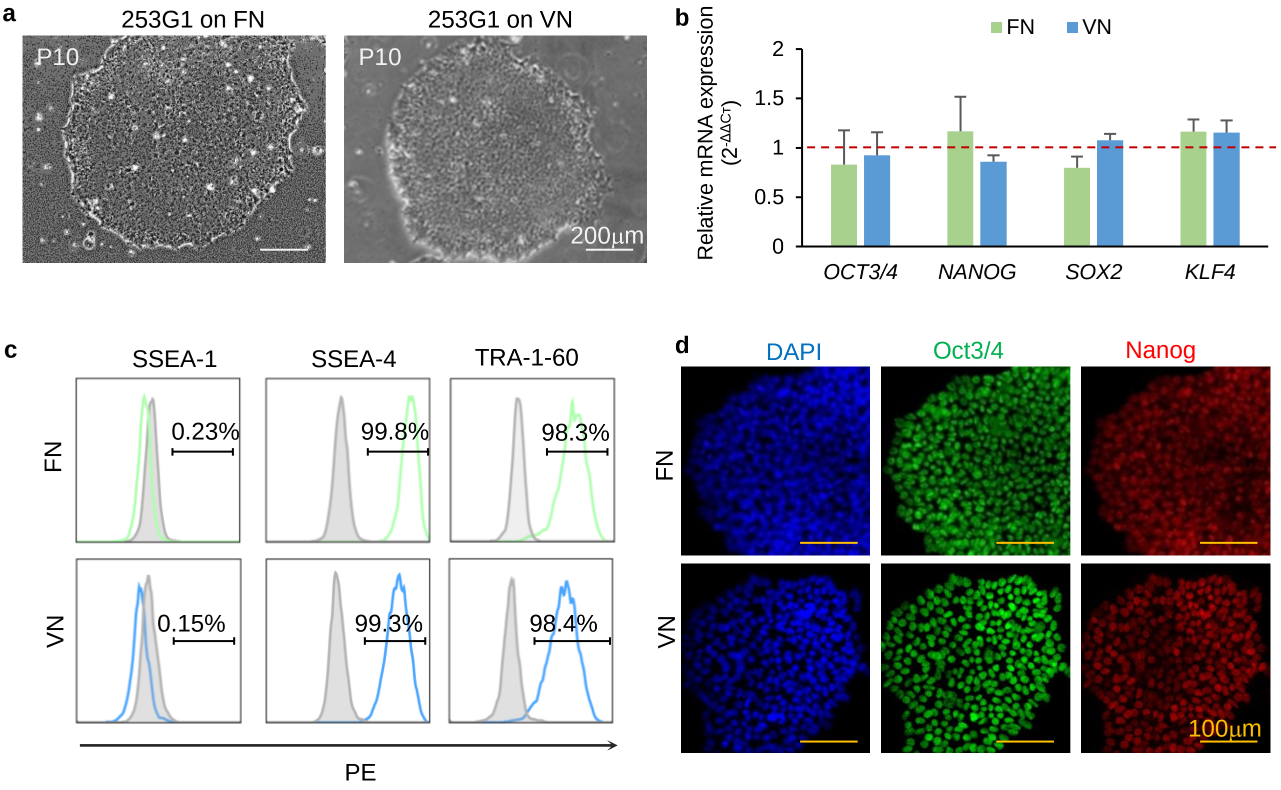

a
253G1 on FN
253G1 on VN
P10
P10
200µm
b
FN
VN
Relative mRNA expression
(2-ΔΔCT)
2
1.5
1
0.5
0
OCT3/4
NANOG
SOX2
KLF4
c
SSEA-1
SSEA-4
TRA-1-60
FN
VN
0.23%
99.8%
98.3%
0.15%
99.3%
98.4%
PE
d
DAPI
Oct3/4
Nanog
FN
VN
100µm

Figure S5

a

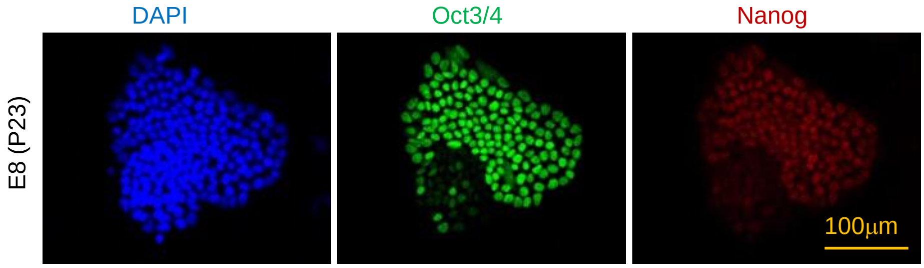

b

Karyotype of 253G1 hiPSCs cultured on iMatrix-511 with E8 (P22)

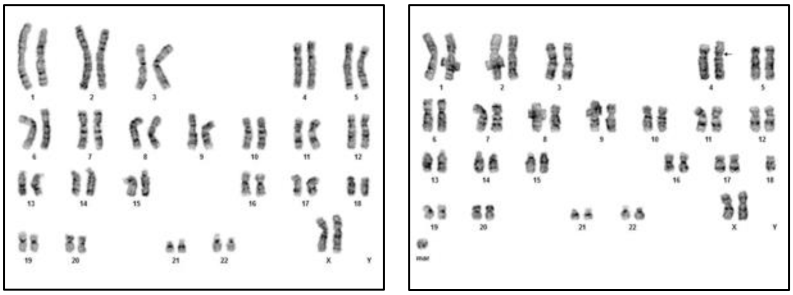

Figure S6

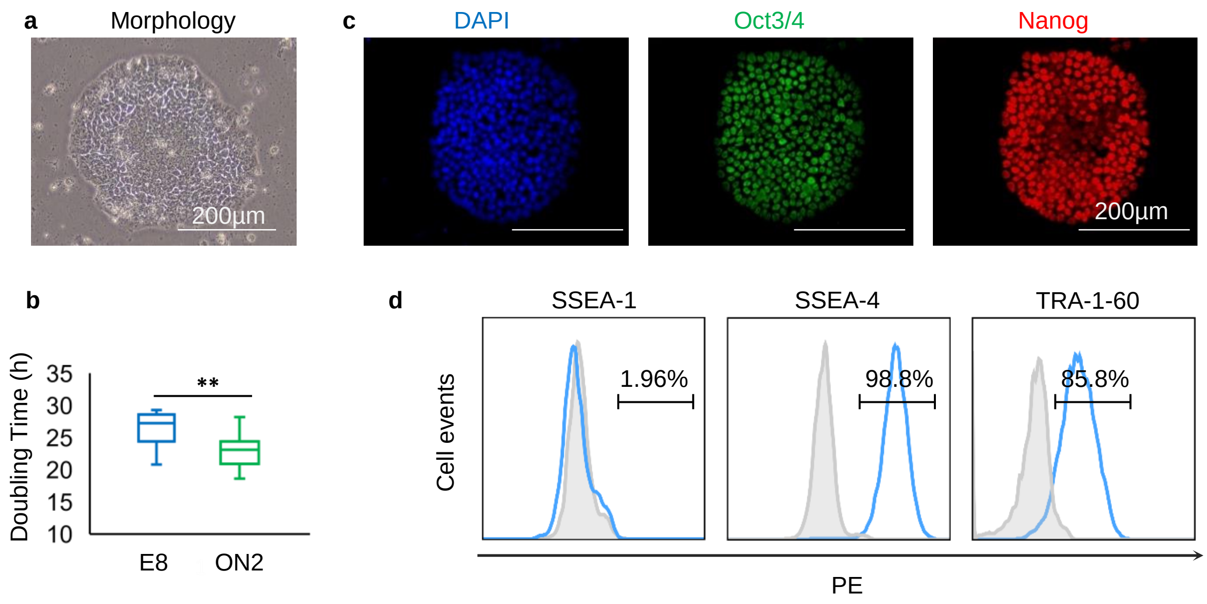

a
Morphology
200μm
c
DAPI
Oct3/4
Nanog
200μm
b
Doubling Time (h)
35
30
25
20
15
10
**
E8
ON2
d
SSEA-1
SSEA-4
TRA-1-60
1.96%
98.8%
85.8%
Cell events
PE
